# Physiological and pathological consequences of exosomes at the blood–brain-barrier interface

**DOI:** 10.1186/s12964-023-01142-z

**Published:** 2023-05-19

**Authors:** Leila Salimi, Fatemehsadat Seyedaghamiri, Mohammad Karimipour, Halimeh Mobarak, Narges Mardi, Maryam Taghavi, Reza Rahbarghazi

**Affiliations:** 1 Department of Applied Cell Sciences, Faculty of Advanced Medical Sciences, Tabriz University of Medical Sciences, Tabriz, Iran; 2 Student Research Committee, Tabriz University of Medical Sciences, Tabriz, Iran; 3 Stem Cell Research Center, Tabriz University of Medical Sciences, Tabriz, Iran

**Keywords:** Blood–brain-barrier, Exosomes, Therapeutic outcomes, Integrity, Neuroangiogenesis outcomes

## Abstract

Blood–brain barrier (BBB) interface with multicellular structure controls strictly the entry of varied circulating macromolecules from the blood-facing surface into the brain parenchyma. Under several pathological conditions within the central nervous system, the integrity of the BBB interface is disrupted due to the abnormal crosstalk between the cellular constituents and the recruitment of inflammatory cells. Exosomes (Exos) are nano-sized extracellular vesicles with diverse therapeutic outcomes. These particles transfer a plethora of signaling molecules with the potential to modulate target cell behavior in a paracrine manner. Here, in the current review article, the therapeutic properties of Exos and their potential in the alleviation of compromised BBB structure were discussed.

Video Abstract

**Supplementary Information:**

The online version contains supplementary material available at 10.1186/s12964-023-01142-z.

## Background

**Fig. 1:**
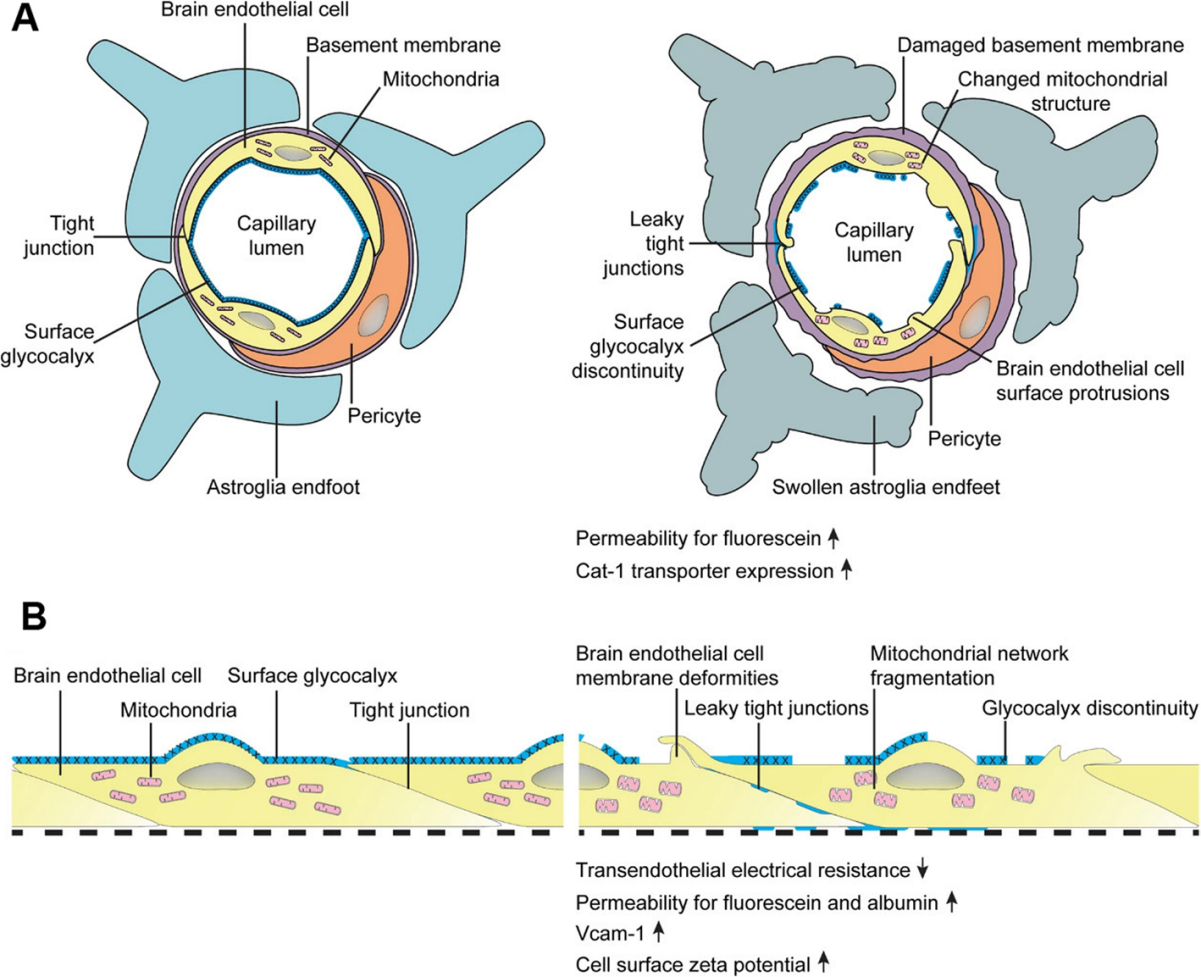
The structure of BBB in *in vivo* **A** and *in vitro* **B** conditions. Brain ECs covers the luminal surface of the BBB interface and are in close contact with blood components. ECs are juxtaposed tightly via the activity of tight junctions, resulting in the formation of a selective barrier interface. ECs are laid on the basal membrane layer which is wrapped by pericytes. Pericytes are sandwiched between ECs at the luminal surface and astrocyte endfeet at the abluminal face. Under pathological conditions, the continuity of the BBB interface is disrupted, leading to the loosening of EC‒to‒EC and EC‒to‒pericyte connections. The production of glycocalyx is not normal on the luminal surface and ECs exhibit several protrusions. Astrocyte endfeet are swollen and lost integrity with pericytes. Along with these changes, the function of several transporters such as cationic amino acid transporter-1 (Cat-1), etc. is disrupted. The increase of adhesion molecules such as VCAM-1 increases the probability of immune cell recruitment and entry via the BBB. The up-regulation of inflammatory cytokines contributes to the abnormal function of tight junction molecules and BBB leakage. Reprinted with permission [18], Copyright 2022. Fluids and Barriers of the CNS

The central nervous system (CNS) is a vascularized system with a close relationship between vascular structure and neurons, indicating the importance of neuro-angiogenesis in the functionality of the CNS [1]. In this regard, a selective border, namely, the blood–brain barrier (BBB), consists of numerous multicellular structures that block the flow of bulky chemicals and immune cells from the circulatory system to the brain [2]. The BBB integrates spatially several cell types to generate functional neurovascular units [1]. Microvascular endothelial cells (ECs) with basal membrane cover the luminal surface while pericytes and astrocytes wrap the abluminal surface [3]. It is suggested that neuronal terminals, as well as microglial cells and localized macrophages, can be juxtaposed with the BBB structure [4]. In the BBB structure, ECs, pericytes, and end-foot astrocytes collaborate to create a natural barrier that limits blood cells traveling to the brain parenchyma [5]. The physiological properties of BBB also regulate the transit of nutrients, chemicals, and medicines from blood to the brain [6]. The phenomenon of transcellular mechanisms or transcytosis mainly relies on the selective interaction of ligands with specific receptors on the luminal surface of endothelial layers [7]. Besides, intracellular metabolic activity can help the inward transport of varied compounds for delivery into the brain parenchyma. BBB not only protects neural tissue from harmful compounds and pollutants in the blood but also constricts the crossing route of therapeutics [8]. Compared to the other tissues, the transfer of varied compounds, especially hydrophilic molecules, is limited through the BBB wall because of the existence of tight junctions between the ECs. The junctional adhesion molecules (JAMs) tightly link the ECs [9]. Productive proteins at tight junctions (TJs) that actively participate in biological interaction include claudins, occludin, and JAMs. Several cytoplasmic factors connect the transmembrane adhesion compounds to the cytoskeleton [10]. TJs are connected to the actin/vinculin-based cytoskeleton and engage with basal endothelium (Fig. 1) [11]. It was thought that BBB is disturbed by conditions like ischemia and other neurological illnesses such as myelin deficiency [12]. These characteristics have the potential to trigger an inflammatory process and oligodendrocyte disruption by causing the continual movement of immune cells from the circulation into the CNS area [13]. Under pathological conditions, microglia can secrete reactive oxygen species (ROS) in response to brain damage or immunological activation, resulting in the disruption of BBB and leakage of plasma into the CNS parenchyma (Fig. 1) [14]. At the BBB level, neurons can interact with astrocytes to control the circulation rate [8]. CNS equilibrium and performance are crucially regulated by the astrocyte's function. Reactive astrocytes are generated in response to CNS damage. Astrocytes can polarize into the neurotoxic or pro-inflammatory phenotype (A1) and the neuroprotective or anti-inflammatory phenotype (A2) [12]. Even though, the straightforward distinction of the A1/A2 phenotypes does not accurately represent the full spectrum of astrocytic phenotypes, it helps us comprehend how astrocytes behave in different CNS illnesses [15]. To this end, specific transport systems have been developed in the BBB interface to regulate the inward and outward movement of molecules between the blood and brain parenchyma. It has been indicated that both paracellular and especially transcellular mechanisms actively participate in the transfer of molecules via BBB interface [1]. The occurrence of neurological pathologies such as multiple sclerosis, encephalitis, Alzheimer’s disease, and ischemic stroke can affect the continuity of the endothelial barrier, leading to several neurological abnormalities [16]. Some parts of the brain harbor the neurovascular units because of the necessity for the perception of fluctuations within the blood and subsequent feedback [17]. Commensurate with these findings, maintenance of cross-talk between the cellular components of BBB under pathological conditions is mandatory to preserve its selective barrier capacity and prevent several neurological deficiencies.

### Exo biogenesis

**Fig. 2:**
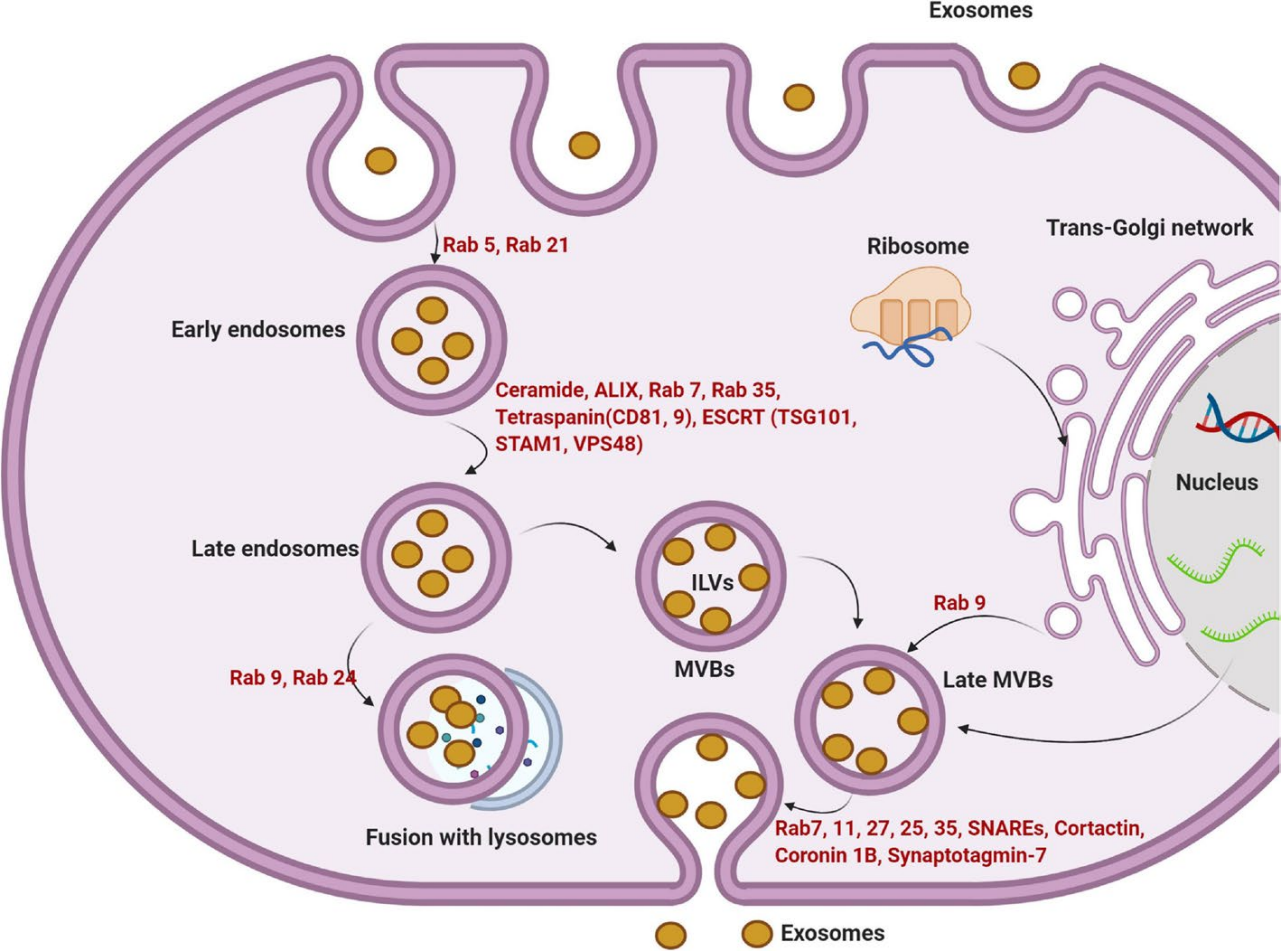
The molecular machinery of Exo biogenesis. Newly generated early endosomes are produced via cell membrane invagination to support the intracellular transmission of uptaken Exos. In the next step, early endosomes mature into later Exos and eventually MVBs. Along with these changes, the invagination of the endosomal membrane into the lumen leads to the formation of several ILVs, known as Exos after secretion into the ECM. The participation of ESCRT independent mechanisms and/or ESCRT complex (ESCRT-0, -I, -II, and -III) with several tetraspanin types is mandatory for the sequestration of new cargo into the ILVs. By engaging the SNARE system and several GTPases, MVBs are directly fused with the cell membrane to release their contents. Exos have nano-sized bi-lipid layer structures with specific molecular identities. ESCRT: endosomal sorting complex transport; MHC: major histocompatibility complex; MVB: multi-vesicular body; Rab: Ras-associated binding proteins. Reprinted with permission [29], Copyright 2021. Frontiers in Cell and Developmental Biology

Among different subclasses of extracellular vesicles (EVs), Exos with an average size of 40–150 nm are secreted into the extracellular matrix (ECM) and actively participate in paracrine interaction between the cells [19, 20]. These nano-sized particles originated from an endosomal system and are involved in varied cellular functions [21]. From a morphological aspect, Exos exhibit a relatively spherical appearance with a lipid bilayer membrane [21, 22]. Molecular investigations have revealed that fundamental components are attached to the lipid membrane in the process of Exo biogenesis inside the cells. For instance, flotillin, several tetraspanin types (CD63, CD9, CD81, and CD82), Alix, tumor susceptibility gene 101 (TSG101), membrane fusion proteins such as RABs, and ARF along with different types of HSPs (HSP60, 70, and 90) can be detected in the abluminal surface of Exo membrane (Fig. 2) [23, 24] Besides, lipid compounds such as ganglioside GM3, cholesterol, sphingomyelin, and ceramides decorate the Exo surface [25]. The existence of these compounds makes the Exo natural shuttles carry the biomolecules between the cells in a paracrine manner [26–29]. Within the cytosol, the procedure of Exo biogenesis is initiated with the early endosome formation. In the latter steps, early endosomes can mature into late endosomes and multivesicular bodies (MVBs) where the numerous intraluminal vesicles (ILVs) are generated via the invagination of the membrane into the lumen. Following the secretion into the ECM, ILVs are known hereafter as Exos [30, 31]. It has been indicated that several types of molecular machinery can expedite the phenomenon of Exo biogenesis by using the endosomal system. Among these effectors, ESCRT machinery is composed of four complexes (ESCRT-0, I, II, and III) and the assistant factor namely AAA ATPase Vps4 has a crucial role in Exo biogenesis. Along with these effectors, ALIX, TSG101, and HRS are touted as ESCRT subunits [32]. ALIX collaborates with the ESCRT-III complex and interacts with the ATG12–ATG3 compound to regulate Exo synthesis. MVB morphology and trafficking can be changed by the inhibition of the ATG12–ATG3 complex [33]. In the ESCRT-dependent pathway, ESCRT-0, I, and II with several ubiquitin-binding domains partake in cargo sorting into the ILVs while the activity of ESCRT-III regulates membrane flexibility, the fission process, and ILVs formation in the lumen of MVBs [34]. It was suggested that the activity of ESCRT-III can be regulated by AAA ATPase Vps4. Unlike ESCRT-dependent mechanisms, tetraspanins such as CD63 and lipids (ceramides) are ESCRT-independent effectors can accelerate the invagination of MVBs membrane and ILV formation [35, 36]. Upon the formation of MVBs, these vesicles can be designated for degradation and fusion with the plasma membrane to release Exos [29]. For lysosomal degradation, the activity of Rab7 is critical while this factor can orchestrate the formation of autophagolysosomes after the activation of autophagic response [31, 36]. To be specific, the Rab-GTPases family including several Rab types are critical components of intracellular trafficking machinery [37]. For instance, Rabs such as Rab35, 11, and 27 in collaboration with SNARE proteins can accelerate the fusion of MVBs with the plasma membrane and Exo release (Fig. 2) [38]. Exo can penetrate target cells by engaging several mechanisms. In direct fusion, the exosomal membrane is merged with the host cell plasma membrane. Receptor-ligand interaction is another strategy that Exo use for internalization. Along with these mechanisms, unspecific phenomena such as clathrin-dependent endocytosis macropinocytosis, micropinocytosis, and lipid raft-dependent have been also detected in terms of Exo internalization [31, 39].

**Fig. 3:**
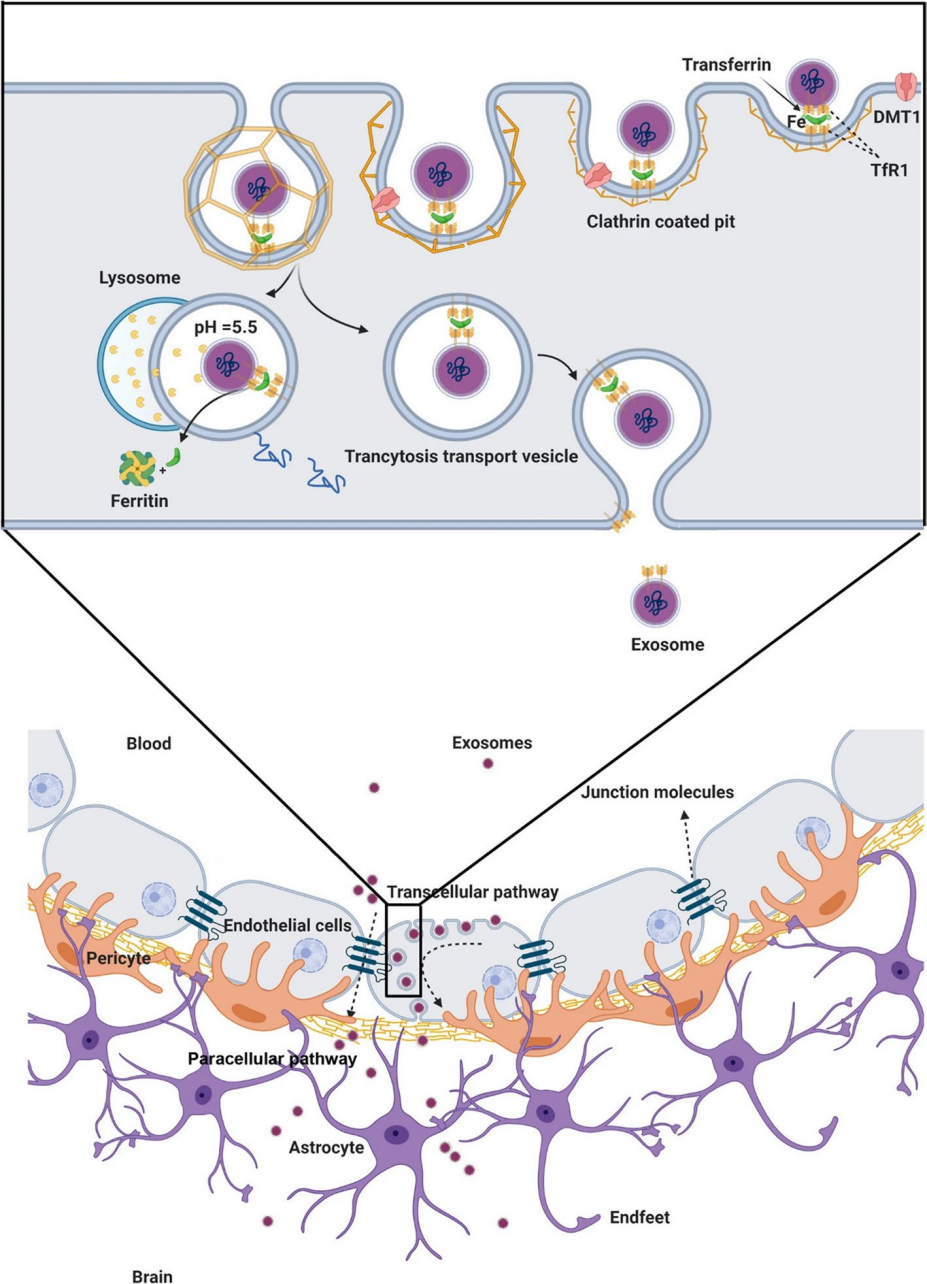
Exos can cross the BBB interface using paracellular and transcellular pathways. Due to the existence of transferrin on the Exo surface, these nanoparticles can attach to the transferrin receptor (TFR) at the luminal surface of brain ECs, leading to the formation of clathrin-coated pits and Exo internalization. The internalized Exos are direct to lysosomal degradation where the released cargo can regulate specific signaling pathways inside the ECs or cross the endothelial barrier and exist through the abluminal surface. After the production of inflammatory cytokines and certain physiological conditions, Exos can cross the BBB via a paracellular route where the function of tight junctions is not abnormal. Reprinted with permission [1], Copyright 2021. Cell & Bioscience

Treatment of neurological diseases has a significant hurdle in the effective transport of macromolecular therapeutic medicines across the BBB to the CNS [40]. Exos have been shown to penetrate the BBB with the help of ECs (Fig. 3). These particles are natural nanomaterials with properties to carry specific genetic and proteomic cargoes to the brain. Besides, the inherent macromolecular transport and targeting characteristics of Exos have been clearly described. Because a load of several compounds is applicable in the Exo surface and lumen, this approach paves the way to cure a variety of neurological diseases by systemic medication transport all over the BBB [41]. It has been shown that bEnd.3 brain endothelial progenitor cells can release Lipocalin-2-loaded Exos from principal brain tumors [42]. Exos also improve bEnd.3 cells membrane permeability in an LCN2-dependent manner via the modulation of the JAK-STAT3 signaling axis. The suppression of LCN2 led to the inhibition of Exo therapeutic effects on BBB ECs. Of note, Exos can act as natural bioshuttles to carry different signaling molecules or regulate the transit of several components through the BBB structure. For instance, it was suggested that the delivery of nanocapsules composed of 2-methacryloyloxyethyl phosphorylcholine, monoclonal antibody, and cross-linker from BBB is closely associated with the activity of released Exos from brain glioma tumor cells. To be specific, the inhibition of Exo using an Akt inhibitor release from tumor mass can efficiently decrease the BBB entry of nanocapsules [43]. Whether cancer Exos can regulate the entry of blood components through BBB should be addressed.

### Exo isolation and enrichment methods

To date, several isolation technologies have been developed for the isolation and enrichment of Exo for biofluids [44]. Because Exo are small-sized particles (40–150 nm) with low density (1.13–1.19 g/ml), the existence of cell fragments and varied protein types may affect the purification of Exo [45]. At present, ultracentrifugation, ultrafiltration, immunoaffinity-based isolation, size exclusion chromatography (SEC), and polymer precipitation are the most common Exo isolation methods in in vitro and in vivo experiments [44]. Near half of the experiments associated with Exo are done via ultracentrifugation methods. This procedure consists of preliminary centrifugation steps at lower speeds, 300, 2000, and 10,000 g to eliminate cell debris and fragments which follows by centrifugations at higher speeds (100,000–120,000 g) for 1–2 h. It seems that the existence of subcellular organelles, infectious viruses, and protein aggregates can reduce the purity of isolated Exo. Besides, Exo isolated by high-speed centrifugation steps exhibited some morphological abnormalities [46].

In ultrafiltration methods, Exo are enriched after passing through filtering membranes with certain pore sizes. The ultrafiltration process is done via engaging forces such as pressure, centrifugation, and electric stimulation [47]. It is thought that Exo and particles smaller than Exo can pass the membranes with permeate. Morphological studies have shown Exo deformation isolated by the ultrafiltration method. Because of Exo absorbance within the membranes, the total number of isolated Exo is less which difficult the application of this method in small volume samples [48].

In the SEC method, biofluids are passed through columns with porous beads and small-sized particles can enter the pores with lower elution speed. Based on the previous data, SEC can yield Exo with high-rate purity like the ultrafiltration technique [49]. The isolation of Exo via immunoaffinity is conducted based on the physical interaction of exosomal antigens (tetraspanins, etc.) with antibodies. For this purpose, antibodies are conjugated with magnetic beads, and chromatography substrates [50]. This technique helps the researchers to fraction the specific types of Exo, *i.e.* cancer Exo from normal counterparts, based on specific exosomal surface antigens [51]. It was suggested that price, genetic instability, and lack of homogeneity after antibody functionalization limit the bulk application of immunoaffinity-based technologies for Exo isolation [52]. The application of a single antibody for Exo isolation can increase the enrichment of the target Exo population but reduce the minimum number of Exo required for biological analyses [51].

Using several polymers such as polyethylene, acetate, protamine, etc. Exo can be precipitated in low centrifugation rates for large sample sizes [53]. In recent years, several isolation kits have been developed for Exo enrichment from biofluids. For instance, “Exosome Isolation kit”, “Eloquence”, and “Exo-spin” are the most common available kits. Despite the ease of use, the Exo values such as purity, number, and size distribution are so variable [54]. Along with approaches, other modalities such as membrane-based separation, microfluidics, and other modalities can be used for the isolation of Exo [55].

### Exo and BBB crossing

The existence of an impenetrable BBB interface limits the exchanges of compounds (400–600 Da <) between the blood and cerebrospinal fluid, resulting in the maintenance of neuronal homeostasis in CNS [56]. In this regard, several molecular mechanisms are engaged to orchestrate the BBB crossing of varied molecules [57]. Several mechanisms such as direct fusion, clathrin- and caveolin-mediated endocytosis, ligand-receptor interaction, macropinocytosis, and phagocytosis are involved in the entry of Exo into the brain parenchyma [58, 59]. Which mechanisms are dominant Exo entry routes under physiological and pathological conditions remained unknown. In an experiment, it was shown that bovine milk eGFP^+^ EVs can be easily uptaken by mouse bEnd.3 cells in an in vitro chamber model and reached BV2 microglia on the bottom surface [57]. Brain microvascular ECs are located at the blood side of the BBB structure and are in close contact with circulating Exos [60]. It has been indicated that Exo can release their content into EC cytosol after direct fusion [61]. It is possible that entering Exo are directed to lysosomal degradation and/or early and late endosomes [62]. Besides, Exo can be transferred to the luminal surface of brain ECs [63]. Of note, the Exo subpopulation less than 50 nm in diameter, known also as exomers, can reach the EC abluminal side compared to large-size Exo (50–150 nm) [64]. Compared to paracytosis, it seems that transcytosis is the main route of Exo entry to brain ECs. In an in vitro Transwell Insert assay, treatment of ECs with an endocytosis inhibitor namely Dynasore prohibited the number of labeled Exo at the abluminal surface, indicating the importance of endocytosis in BBB crossing of circulating Exo [59].

Several data have indicated the existence of clathrin pits on the luminal surface of brain EC which are involved in clathrin-based transcytosis [65]. Upon the attachment of Exo ligands to cognate receptors, clathrin vesicles containing both receptors and ligands are generated and can deliver their contents at the abluminal surface [66]. For example, exosomal tetraspanins can interact directly with surface integrins such as integrin α4 [67]. It was indicated that the interaction of macrophage Exo LFA-1 with endothelial ICAM-1 promotes Exo diapedesis across the BBB [68]. Other receptors such as the transferrin receptor (TfR) are also involved in the delivery of Exo into brain microvascular ECs and BBB crossing via the dynamic endocytosis cycle [69]. These data indicate that multiple molecular mechanisms may participate in the BBB crossing of Exo. Further investigations are highly recommended to address the exact role of each entry route under physiological and pathological conditions.

### Anti/inflammatory properties of Exos

Inflammation is a natural biological process that occurs in response to several pathological conditions to reestablish tissue homeostasis (Table 1) [70]. Neuroinflammation is a common hallmark reported under neurodegenerative conditions such as Alzheimer’s disease (AD), amyotrophic lateral sclerosis (ALS), etc. [71]. Under inflammatory conditions, the stimulation of glial cells can lead to the local or extensive elevation of pro-inflammatory cytokines within the brain parenchyma. If the host tissue is not able to remove the stimuli or the inflammatory response continues over long periods, uncontrolled inflammatory reactions cause injuries [72].

**Table 1:** The role of inflammatory factors in neurodegenerative diseases

Neurodegenerative diseases	Neuronal cargo	Activity	Inflammatory factors	Ref
**Alzheimer's disease (AD)**	neurofibrillary tangles (NFT) and amyloid-beta peptide (Aβ)	Aggregates of hyperphosphorylated tau lead to ROS production and inflammatory responses	IL-1, IL-6, TNF-α, and PGE2	[73]
**Parkinson’s disease (PD)**	alpha-synuclein (α-syn) leads to the loss of dopaminergic neurons	Control of neurotransmitter release, through effects on the SNARE complex	C-reactive protein (CRP), IL-6, and TNF-α	[74]
**Amyotrophic lateral sclerosis**	p75ECD phosphorylated neurofilament heavy (pNfH) neurofilament light (NfL)	Involved in neuroprotective actions against Aβ toxicity	CRP, IL-6, IL-8, TNF-α, IL-1β, IL-17, IL-33, IL-10, Monocyte chemoattractant protein 1 (MCP-1), and IFNγ	[75]
**Huntington's disease**	rab11 activity	Affects the recycling of transferrin receptor and neuronal glutamate/cysteine transporter EAAC1	IL-1, IL-6, TNF-α, TGF-β	[76]
**Lewy body disease**	alpha-synuclein	localizes specifically to the nerve terminal and inhibits neurotransmitter release when over-expressed	IL-1β, TNF-α, IL-6, and IL-10	[77]
**Multiple sclerosis (MS)**	neutrophil-to-lymphocyte ratio	oligodendrocyte damage and demyelination	IL-6, IFN-γ, TNF-α, and granulocyte–macrophage colony-stimulating factor (GM-CSF)	[78]

**Fig. 4:**
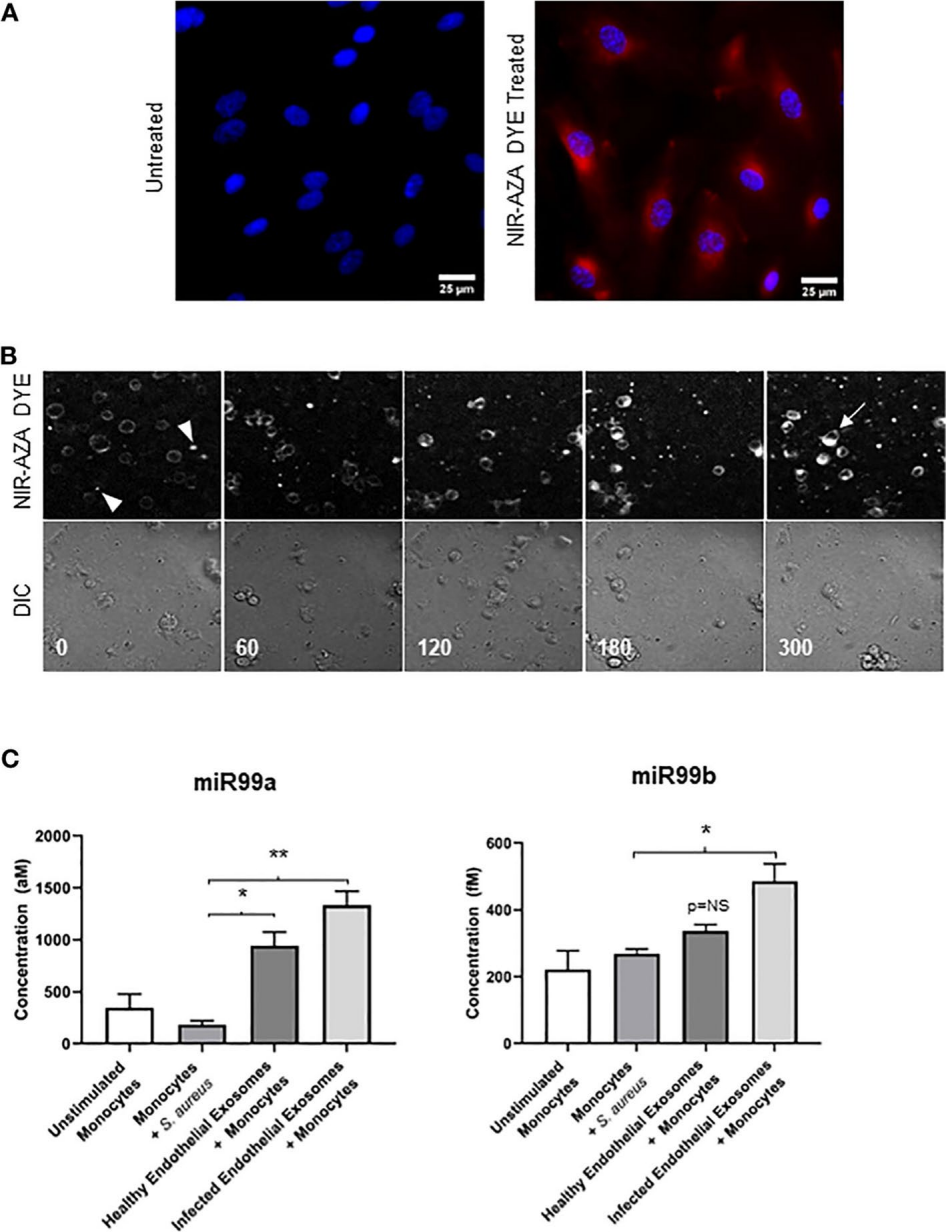
Effect of inflamed EC Exos on THP-1 monocytes **A-C**. Human aortic ECs were incubated with Near-Infrared BF2-azadipyrromethene (NIR-AZA) dye (red). Nuclei were stained with DAPI **A**. THP-1 monocytes were incubated with Exos isolated from normal and inflamed ECs inside the ibidi chambers and imaged every 5 min over 16 h [time-lapse across 300 min] **B** [top panel: Cy5 channel, bottom panel: DIC channel]. At time 0, no detectable fluorescence signals can be achieved indicating the lack of interaction between Exos and monocytes (arrowheads). The intensity of fluorescence signals reached the maximum levels after 300 min. The existing materials inside the cells indicate the successful uptake of Exos (arrow). Real-time PCR analysis **C**. The direct exposure of monocytes to *Staphylococcus aureus* did not yield statistically significant differences in terms of miR-99a/b. The incubation of monocytes with normal Exos up-regulated the expression of miR-99a as compared to the non-treated monocytes. Inflamed Exos isolated from ECs contributed to the higher levels of miR-99a and miR-99b in monocytes. These data indicate the uptake of introduced Exos by monocytes (*n* = 3); One-Way ANOVA; *p < 0.05, **p < 0.01; NS: not significant. Reprinted with permission [82], Copyright 2022. Frontiers in Cellular and Infection Microbiology

**Fig. 5:**
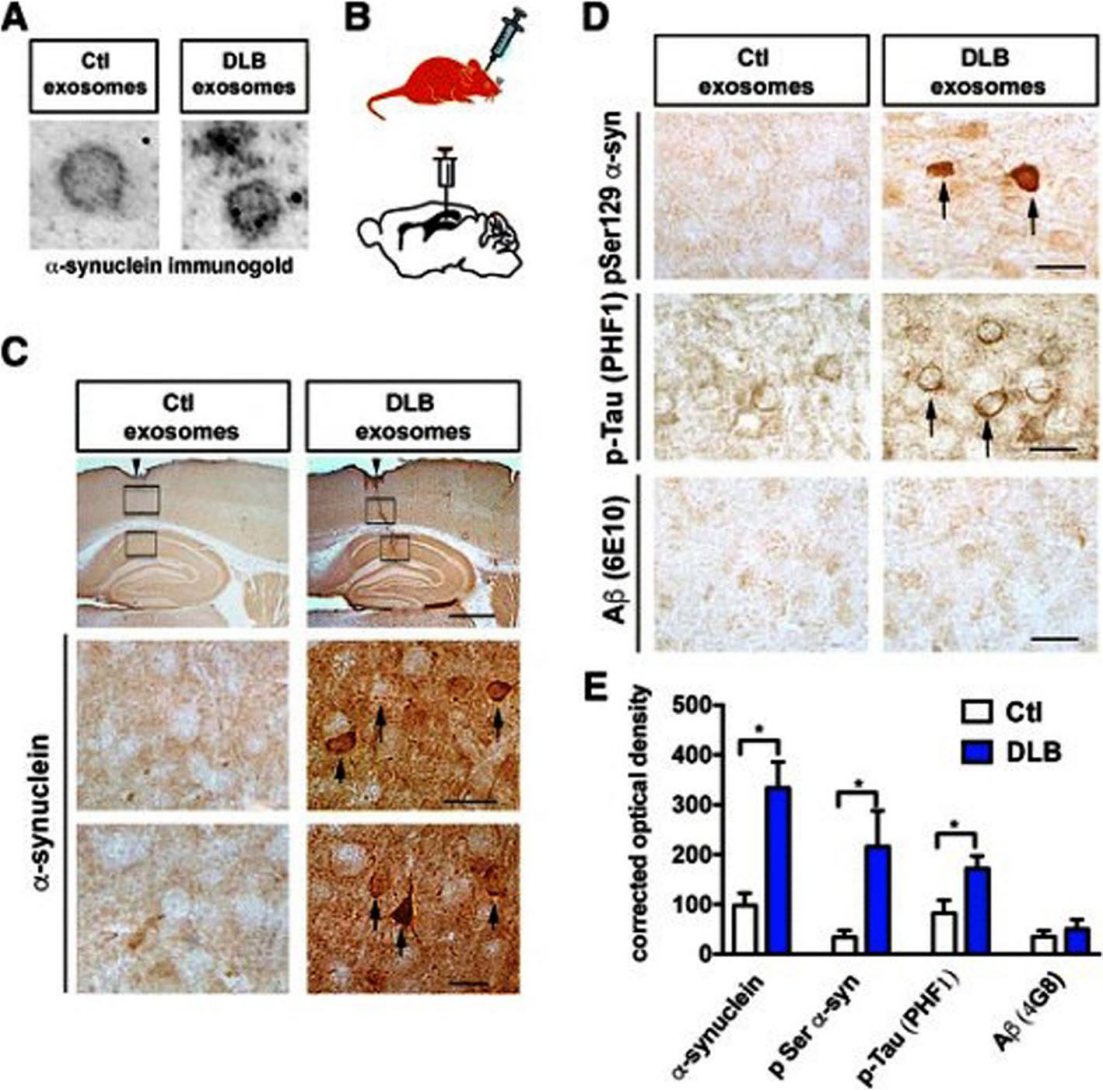
Monitoring the effect of dementia with Lewy bodies patient Exos on intracellular accumulation of phosphorylated proteins. Detection of exosomal α-synuclein using immuno-gold labeling and TEM images (Ctl: Control; DLB: dementia with Lewy bodies patient) **A**. Stereotaxic injection of prepared Exos into C57BL/6 N × DBA/2 F1 mouse hippocampus **B**. Bright-field imaging of brain slides after injection of Exos **C** [row 1: Sagittal view of the hippocampus. The site of needle injection is highlighted by arrowheads (Scale bar: 150 μm). Arrows indicate immuno-labeled cell bodies (Scale bar: 25 μm). Brain samples were stained using anti- phosphorylated α-synuclein (pSer129), PHF1 and Aβ (6E10) [(Scale bar: 25 μm]. Semi-quantification of immunohistochemical stains using an optical density. *p < 0.05. Reprinted with permission [85], Copyright 2017. Acta Neuropathologica Communications

**Fig. 6:**
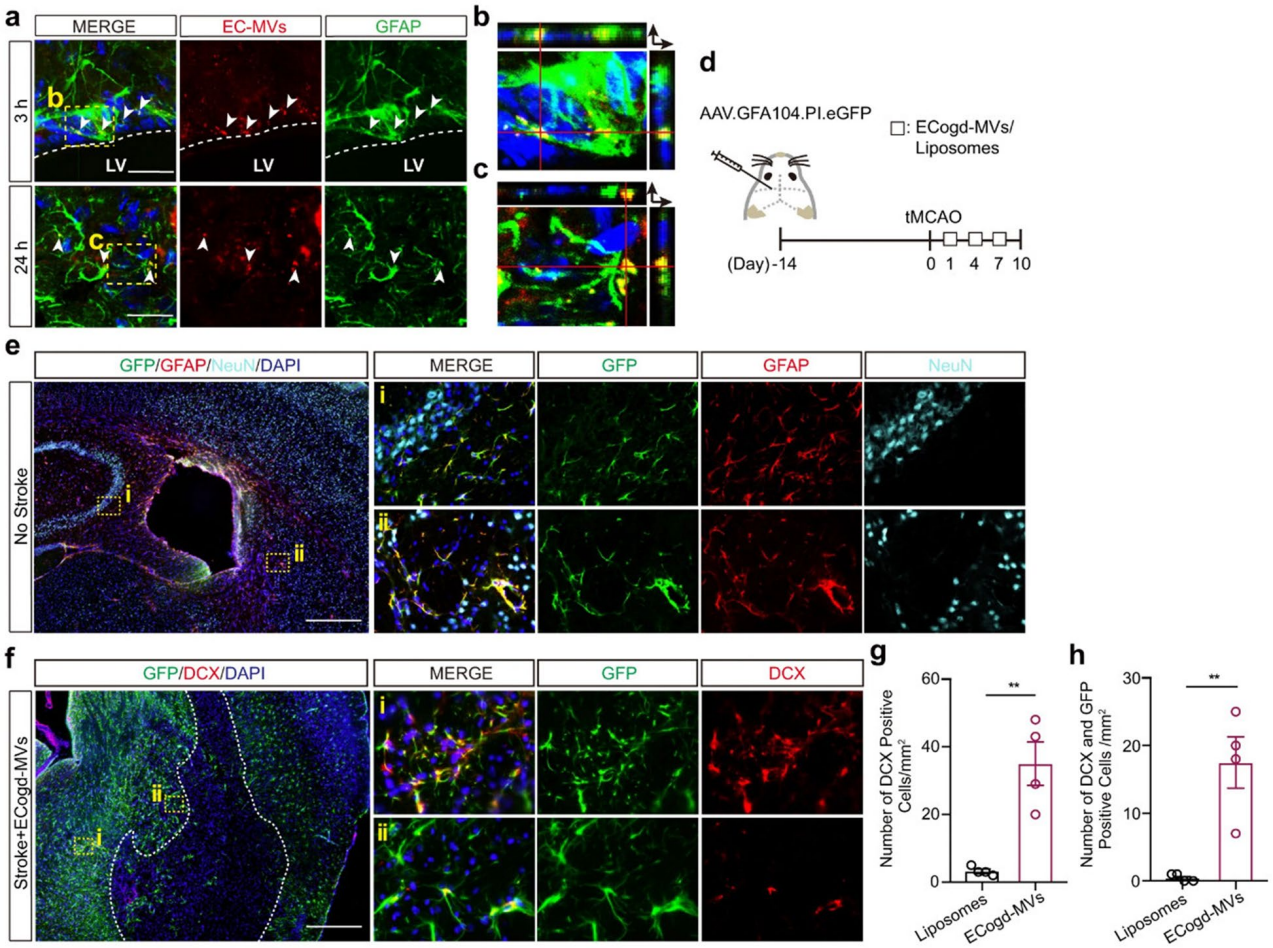
Hypoxia and hypoglycemia-treated ECs produce microvesicles (EC-MVs) with the potential to induce astrocyte differentiation toward neural progenitor cells in a mouse model of ischemia (**a**-**h**). Confocal images revealed the successful entry of red color Cy3-labeled EC microvesicles (EC-MVs) by astrocytes after 3 (**a**, **b**; Scale bar: 25 μm) and 24 h (**a**, **c**; Scale bar: 25 μm) after injection into the lateral ventricle (White color dash lines: lateral ventricle border). Schematic of experimental procedure (**d**). The expression of GFP, GFAP, and NeuN 14 days after administration of AAV.GFA104.PI.eGFP vector (**e**; Scale bar: 200 μm). In panel **f**, GFP and DCX positive cells were monitored on day 10 with transient MCAO (Scale bar: 200 μm). The number of DCX^+^ neurons/mm^2^ was measured in the peri-infarct zone (**g**) ROIs were calculated per each Sect. (3 sections per animal; each in 4 mice; *p* = 0.0026). DCX^+^/GFP^+^ cells per mm^2^ of in peri-infarct zone (3 sections per animal; each in 4 mice; *p* = 0.0043) (**h**). Three (**a**) and four (**e**, **f**) sets of experiments were performed in this study. Abbreviation: Doublecortin: DCX; Glial fibrillary acidic protein: GFAP; Green fluorescent protein: GFP; Neuronal nuclei: NeuN; Regions of Interests; ROIs. Reprinted with permission, [94] Copyright 2022. Nature Communications

Exos are practically able to transport biomolecules and signaling payloads to different targeting cells [28]. Unlike synthetic nanoparticles, Exos are relatively stable vesicles without toxicity and can cross several biological barriers such as BBB [1, 26]. Due to their potential to carry several biomarkers associated with diseases such as AD [β-amyloid (Aβ), tau] and Parkinson's disease, (α-synuclein), etc., Exos are considered important biological elements for early-stage detection and monitoring of pathological conditions [79]. Notably, microglia-related Exos harboring tau and Aβ can transmit pro-inflammatory signals to remote sites [80]. Several aspects uncovered that Exos can transfer specific biomolecules which are identical to certain pathological conditions [22]. Molecular identification of exosomal cargo has revealed the existence of inflammatory modulators such as inflammatory miRNAs and varied relevant proteins with the potential to act in close and remote sites [81]. In an experiment conducted by Fitzpatrick et al., it was indicated that Exos isolated from *Staphylococcus aureus* exposed human aortic ECs increased the expression of CD11b and MHCII in THP-1 monocytes [82]. Based on the data, exosomal transfer of miR-99a and miR-99b inhibits the mTOR activity in monocytes, resulting in increased production of IL-6, and reduction of IL-10 (Fig. 4) [82]. These findings are consistent with the fact that direct exposure of BBB ECs to sepsis and inflammatory conditions can contribute to the release of Exos with certain signaling molecules that recruit immune cells and affect the integrity of the BBB interface. To be specific, the exposure of brain ECs to infectious agents and noxious compounds supports the production and release of certain Exo types that lead to the worsening of pathological conditions. Of note, the occurrence of adaptive immune system response with regulated inflammatory cascades can alleviate brain injuries [83]. Within the CNS, the transfer of α-synuclein, Aβ, and prions account for the distribution of these biomarkers between the cells, resulting in the dissemination of inflammatory cascades [84]. Ngolab and co-workers indicated that the injection of Exos from Dementia with Lewy Bodies patients into the murine brain parenchyma promotes α-synuclein aggregation in MAP2^+^, Rab5^+^ neurons (Fig. 5) [85]. Thus, one could hypothesize that Exo functions as disease-promoting or restraining modulators, and these features are closely associated with the entity of exosomal cargo and cell state [84]. Besides, examining the content of Exos in several CNS injuries with different severity of cell damages can help us to predict the side effects or protective properties of these particles. It has been shown that exosomal miR-124-3p can lead to the suppression of inflammatory response within the nervous system and induction of neurite formation. From the molecular aspect, this miRNA regulates M1 inflammatory cytokines via engaging the PI3K/Akt/NF-κB axis [86]. Besides, miR-124-3p protects brain microvascular cells via the promotion of autophagy [87]. Therefore, the induction of autophagy by Exos is touted as an alternative supporting mechanism for cell survival during pathological conditions [29]. Due to the existence of heterogeneous Exos in the circulation system, it is postulated the direct contact of these particles in the bloody face of BBB can regulate the EC activity via the modulation of distinct signaling pathways [88]. In support of this notion, the injection of healthy rat serum Exos diminished the infarct volume via the reduction of apoptotic changes in BBB ECs in rats with ischemic stroke [88]. Protein levels of ZO-1 and Claudin-5 were induced and coincided with the reduction of MMP-9 and Evans blue leakage to the affected sites, highlighting the restoration of BBB continuity. Consistent with these data, the excessive autophagic response was indicated by reduced LC3-II/LC3-I ratio and autophagosome number in bEnd.3 cells after being exposed to oxygen–glucose deprivation [88]. The inhibition of autophagic response using chemical compounds like 3-methyladenine blunted uncontrolled autophagic activity and degradation of occludins in a rat model of stroke [89]. On contrary, Yang and co-workers indicated that the stimulation of autophagy in zebrafish can restore the function of BBB ECs via recycling aggregated claudin 5 distributed by caveolin 1 related mechanisms [90]. Whether the activation or inhibition of autophagy can restore the function of BBB and ECs needs further investigations. Any interpretations should be in accordance with the stage of pathological conditions and the intensity of autophagy response [91]. It confirmed that the source of Exos from different cellular source after the promotion of brain injury is integral to BBB function and continuity [92]. In addition to the effect of glia Exos on brain ECs, emerging data have pointed to the fact that EC Exos can exert neuroprotective effects on injured neurons and other cell types [93]. In an in vitro co-culture system using human umbilical cord ECs and primary mouse hippocampal neurons, the apoptotic changes were diminished in neurons exposed to hypoxic and hypoglycemic conditions. Data indicated that hypoxic neurons can uptake EC Exos via caveolin-1 based endocytosis, leading to the alteration of miR-1290. The inhibition of Exo secretion via chemical inhibitor namely GW4869 from ECs led to the promotion of apoptosis rate in injured neurons [93]. These data indicate the crucial role of brain microvascular ECs in the neuroprotection in a paracrine manner. Whether the release of Exos by ECs precedes the brain injury or is done in response to the signaling molecules should be addressed. The activation of Exo production is possibly a compensatory mechanism to prevent the extension of pathological conditions to other brain sites like BBB interface and to normalize the excessive behavior of glial cells after being exposed to injured neurons. Interestingly, EC can shed Exos with a pro-neural factor namely Ascl1 under the ischemic stroke. The entry of these Exos in astrocytes can intensify astrocyte trans-differentiation into neural progenitors (Fig. 6) [94]. These features show the vascular cell-based regulation of neuroplasticity via the secretion of Exos under pathological conditions. This phenomenon occurred at the levels of certain factors transferred by Exos inside the astrocytes in a paracrine manner.

The source and metabolic status of parent glial cells are important in the production of Exos affecting the BBB interface [95, 96]. For instance, recent works have indicated that inflamed astrocyte-derived Exos harbor large amounts of IL-1β and HMGB1 with the potential to induce an inflammatory response in the target cells [49, 97]. In support of this statement, the incubation of human brain ECs with AD mouse astrocyte EVs led to the reduction of electrical resistance, and down-regulation of tight junction proteins such as claudin-5, occludin, and ZO-1 when compared to the healthy astrocytes [98, 99]. Commensurate with these comments, the differential contents of Exos from varied cell types may contribute to the suppression and/or induction of inflammation in vascular cells within the BBB structure during pathological conditions [22].

### Angiogenic and arteriogenic properties of Exos

The formation of de novo blood vessels is defined as angiogenesis or neo-angiogenesis [100]. The lack of blood nourishment can contribute to ischemic changes. Therefore, the promotion of angiogenesis seems critical issue following ischemic stroke [100]. Several studies have proved the eligibility of Exos in the promotion and inhibition of angiogenesis in either in vitro or in vivo conditions (Table 2) [101, 102]. It has been indicated that stem cells and mature cell types can release angiocrine via the secretion of Exos [103–106]. Exos may transport a wide range of macromolecules from origin cells to acceptor cells, including microRNAs, nucleotide sequences, proteins, lipids, and even psychoactive agents [107]. Exos can control the intracellular levels of angiogenesis-related RNAs and proteins after the induction of ischemic response. For example, it was suggested that exosomal cargo increases the expression of VEGF, leading to diminished infarct size [108]. Upon the attachment of VEGF to tyrosine kinase VEGFR-2, the formation of new blood and lymphatic vessels is initiated [109]. Along with these changes, the endothelial basal membrane is disrupted by the activity of MMP-2 and -9 to accelerate ECs migration in the direction of cytokines and proangiogenic factors [110]. The promotion of angiogenesis coincides with the induction of permeability in the endothelial layer [111]. In an experiment conducted by Liu and co-workers, they proved that brain EC monolayer integrity was restored in 5 × FAD mouse (animal model applicable to AD) after exposure to neural stem cell Exo in a Transwell insert system [112]. Yet, the influence of adaptive and excessive angiogenesis during pathological conditions in terms of BBB continuity remains unknown. Irrespective of being adaptive or pathological angiogenesis response, the local or systemic elevation of angiogenic factors can disrupt the EC‒to-EC integrity because of the production of pro-angiogenesis factors like VEGF, etc. [113]. To be specific, the tightly regulation of pro-angiogenic factors at early stages after angiogenesis initiation is mandatory to control BBB leakage and unwanted outcome in response to varied pathological conditions [113]. Higher VEGF levels in ischemic conditions led to the destruction of basal lamina (IV type collagen), activation of MMP-2, and -9, and abnormal BBB leakage [113]. As a correlate, the angiogenic properties of Exos in the alleviation of compromised BBB structure should be interpreted logically. Following ischemic stroke, the elevation of miRNA212 and miRNA132 in brain ECs and exosomal levels of these miRNAs blunts the angiogenic levels and reduces the barrier continuity via the suppression of claudin-1, junctional adhesion molecule 3, and tight junction-associated protein 1 [114]. Noteworthy, it would be possible that the changes in Exo cargoes during pathological conditions within the brain parenchyma and other tissues. Therefore, it seems that the intensity and type of angiogenic response can be different in brain ECs after exposure to Exos from normal and injured or inflamed cells.

**Table 2:** The role of Exo in the diagnosis, therapy, and function in angiogenesis

Exosomal cargo	Mechanisms	Target cell	Function	References
**Annexin A2**	Making of plasmin	Macrophages and ECs	Pro-angiogenesis	[115]
**Angiopoietin-2**	Tie2-unrelated route	HUVECs	Pro-angiogenesis	[116]
**Alcohol acetyltransferase II, Metastasis Associated 1, Seryl-TRNA Synthetase 1, Rho-associated coiled-coil containing protein kinase 1/2**	Increased VEGF and HIF-1 expression	HUVECs	Pro-angiogenesis	[117]
**Carbonic anhydrase 9**	Enhancing the transcription of MMP-2	HUVECs	Pro-angiogenesis	[118]
**SRC Proto-Oncogene, Non-Receptor Tyrosine Kinase, insulin-like growth factor 1 receptor, Focal adhesion kinase**	VEGF overexpression	Endothelial cells	Pro-angiogenesis	[119]
**The cluster of differentiation 147**	increase in VEGF and MMP	HUVECs	Pro-angiogenesis	[120]
**Delta Like Canonical Notch Ligand 4**	Stopping notch indication	U373 and HUVECs	Pro-angiogenesis	[121]
**EGF Like Repeats And Discoidin Domains 3**	encourage cell migration and expansion		Pro-angiogenesis	[122]
**Epidermal growth factor receptor III**	By initiating the MAPK and Akt paths, the interpretation of the VEGF gene is increased	U373 and HUVECs	Pro-angiogenesis	[123]
**Family With Sequence Similarity 225 Member A lncRNA**	By disappointing its receptors NETO2 and FOXP1 as a result of miR-206 mooching, the PI3K/Akt/NF-B/Snail axis is activated	HUVECs	Pro-angiogenesis	[124]
**High Mobility Group Box 3**	In vivo and in vitro, nEXOs that included HMGB3 sped up vasculature	HUVECs	Pro-angiogenesis	[125]
**Influence of interleukin-8**	Regulation via MAPK	HUVECs	Pro-angiogenesis	[126]
**Intercellular Adhesion Molecule 1, CD44v5**	p38 MAPK, RhoA/ROCK, ERK1/2 kinase, Src kinase, and eNOS	HUVECs	Pro-angiogenesis	[127]
**lncRNA UCA1**	The mechanism for AMOTL2/ERK1/2 Trafficking	HUVECs	Pro-angiogenesis	[128]
**IL-6, VEGF, and MMP-2**	Route for WNT5A signal	Endothelial cells	Pro-angiogenesis	[129]
**IL-8, PDGF**	PI3K/AKT Trafficking	Endothelial cells	Pro-angiogenesis	[130]
**GM-CSF, HIF1α, HIF-2α**	Increased VEGF transcription	ECs and M1/M2 macrophages	Pro-angiogenesis	[131]
**miR-148a-3p**	Constraining ERRFI1 to activate the EGFR/MAPK transcription factors	HUVECs	Pro-angiogenesis	[132]
**miR-373**	β-catenin/ Wnt Trafficking	Endothelial cells	Pro-angiogenesis	[133]
**miR-155**	VEGF-A expression is increased through the VHL/HIF-1 route	ARPE-19	Pro-angiogenesis	[134]
**miR-182-5p**	concentrating on Elements 2 and 4 as Kruppel	HUVECs	Pro-angiogenesis	[135]
**miR-9**	JAK-STAT mechanism	Endothelial cells	Pro-angiogenesis	[136]
**miR-497**	VEGF and HIF-1 production are both downregulated	HUVECs	Anti-angiogenesis	[137]
**miR-135b**	Elimination of FIH-1	Endothelial cells	Pro-angiogenesis	[138]
**miR-141**	miR-141/KLF12 passageway in Exos	HUVECs	Pro-angiogenesis	[139]
**miR-10b**	reduction in the amount of HOXD10 and KLF4 proteins	HUVECs	Promotes cell invasion	[140]
**miR-145**	STIM1 stimulates vasculature by inhibiting the IRS1-targeting exosomal miR-145	HUVECs	Pro-angiogenesis	[141]
**miR-21**	VEGF overexpression	HUVECs	Pro-angiogenesis	[142]
**miR-27a**	MiR-27a-loaded Exos from RCCC promote tumorigenesis and suppress SFRP1 transcription	HUVECs	Pro-angiogenesis	[143]
**miR-92a-3p**	KLF2 is the target of exosomal-released miR-92a-3p, which controls morphogenesis	HUVECs	Pro-angiogenesis	[144]
**miR-122**	a reduction in PKM	Normal cells in the pre-metastatic niche	Promotes metastasis, before angiogenesis	[145]
**miR-210**	VEGF overexpression	Endothelial cells	Pro-angiogenesis	[146]
**miR-1290**	SMEK1 is targeted by miR-1290 to produce an angiogenesis phenotype	HUVECs	Pro-angiogenesis	[147]
**miR-549a**	Exosomal miR-549a inhibits HIF1 in HUVECs, which has an impact on the vasculature and endothelial mobility	HUVECs	Pro-angiogenesis	[148]
**miR-21**	enhancing M2 polarity signal transcription in TAMs	CD14 + human monocytes	Pro-angiogenesis	[31]
**M-phase-related transcripts**	Stimulation of cellular proliferation and modification of the M-phase of the cell growth	Endothelial cells	Initiate angiogenesis	[36]
**miR-23a**	By attacking prolyl, exosomal miR-23a promoted vasculature and dilation of blood vessels. adhesion molecules protein ZO-1 and hydroxylase	HUVECs	Pro-angiogenesis	[149]
**miR-181a**	Exos released by the hypoxic PTC carried miR-181a, which inhibits DACT2 by decreasing the expression MLL3 and causes YAP-VEGF-mediated vasculature	HUVECs	Pro-angiogenesis	[150]
**miR-221-3p**	Exos produced from CC cells that contained miR-221-3p increased MVEC vasculature in CC via lowering MAPK10	MVECs	Pro-angiogenesis	[31]
**miR-210**	Diminishment of EFNA3	Endothelial cells	Pro-angiogenesis	[151]
**miR-130a**	reduction of c-MYB	HUVECs	Pro-angiogenesis	[36]
**miR-21**	Rho decreased the expression	HUVECs	Anti-angiogenesis	[152]
**miR-141-3p**	NF-B and JAK/STAT3 communication mechanisms activation	HUVECs	Pro-angiogenesis	[153]
**Neuraminidase**	Overexpression of MMP-9 and CXCR4	786–0	Enhance migration and invasion	[154]
**Neuraminidase**	Src route	HUVECs	Pro-angiogenesis	[155]
**Neuraminidase**	VEGF production is upregulated, whereas hepaCAM transcription is downregulated	HUVECs	Pro-angiogenesis	[31]
**PTCH 1, SMO, SHH, Ihh**	Exos from CC cells stimulate pro-angiogenic responses in endothelium by upregulating Hh-GLI signaling and modifying target genes for angiogenesis upstream side	HUVECs	Pro-angiogenesis	[156]
**Profilin 2**	In H446 and ECs, t PFN2 stimulated Smad2/3 and pERK	HUVECs	Pro-angiogenesis	[31]
**PFKFB-3**	The development of lactose and Fru-2,6-P2 is rising	HUVECs	Pro-angiogenesis	[157]
**RAMP2-AS1 lncRNA**	By depressing miR-2355-target 5p's VEGFR2 by miR-2355-5p syphoning, angiogenesis cell membrane receptors are increased	HUVECs	Pro-angiogenesis	[31]
**TIE2**	Exo-mediated induction of TIE2-expressing macrophages by TIE2-high cancer cells occurs	HUVECs	Pro-angiogenesis	[158]
**TGF-β**	Transmission reliant on SMAD	Fibroblasts	Pro-angiogenesis and pro-tumorigenesis	[159]
**Tetraspanin Tspan8 (D6.1A)**	MMP, VEGF, and VEGFR transcription is increased	Endothelial cells	Pro-angiogenesis	[160]
**VEGF**	uses its tyrosine kinase domains to effect action	HUVECs	Pro-angiogenesis	[31]
**VEGF-A**	The increased angiogenic capacity of brain ECs	Brain microvascular Endothelial cells	Pro-angiogenesis	[22]
**Vasorin**	encourage cell migration and expansion	HUVECs	Pro-angiogenesis	[161]
**Wnt4**	β-catenin/ Wnt circuit	Endothelial cells	Pro-angiogenesis	[22]

**Fig. 7:**
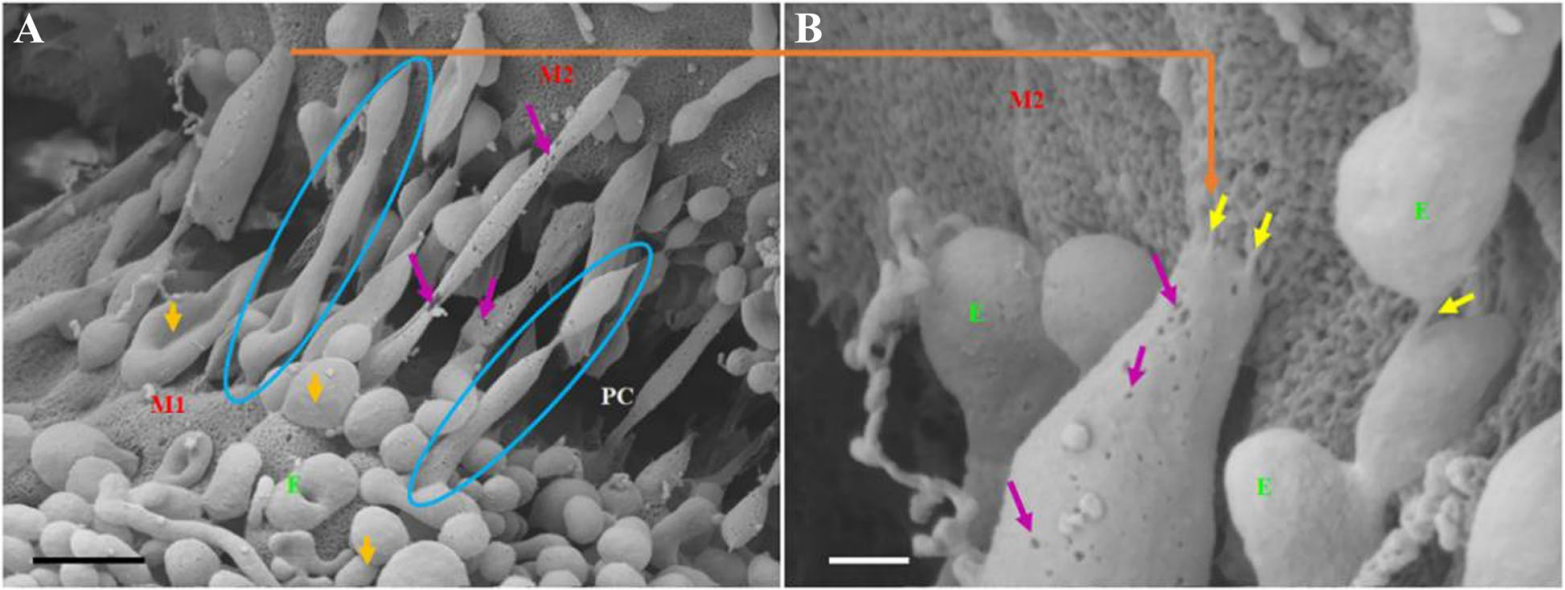
Formation of long tunneling nanotubes between brain ECs to transfer Exis. Exos can be transferred to adjacent ECs via the formation of long, hollow tubes (**A**; Scale bar: 1000 nm). Magnification of target area **B**. Tunneling nanotubes with sticky ends attach to juxtaposed cell surfaces (Scale bar: 200 nm). M1: propose membrane of cells with originating tunneling nanotubes; M2: target cell membrane interacted with tunneling nanotubes from other cells. PC: paracellular space between juxtaposed brain ECs. Blue circles: tunneling nanotube formation; Purple arrow indicates several pores on the membrane of tunneling nanotubes. Yellow arrows indicate the interaction between tunneling nanotubes and target BCs at the extremities. Reprinted with permission [167], Copyright 2021. Frontiers in Neuroanatomy

Among several cell types, the paracrine activity of stem cells has been documented in terms of angiogenesis and tissue healing [162]. Gao and co-workers indicated that injection of endothelial progenitor cell (EPC) Exos via the tail vein can restore the function of injured ECs in mouse traumatic brain injury [163]. Histological analysis revealed the successful uptake of PKH67-labeled Exos by brain ECs coincided with the reduction of brain edema. In the acceptor cells, the levels of PTEN and phosphorylated Akt were reduced with the increase of occludin and ZO-1 [163]. Because EPCs can be recruited into the brain after cerebrovascular damage, it is logical to mention that Exos from EPC sources are valid therapeutic tools to restore BBB integrity. Of note, progressive loss of homeostasis in EPCs can affect the therapeutic effects of ECs [164]. In a study, it was confirmed that pre-treatment with TNF-α can trigger apoptotic changes in EPCs. Incubation of brain ECs with Exos from apoptotic EPCs led to significant oxidative stress and expression of miR126 and eNOS compared to Exos isolated from starved EPCs [164]. Besides the direct effect of EPC Exos on brain ECs, astrocytes are other target cells with the ability to uptake regenerative Exos [165]. In in vivo conditions, ECs are frontline cells to uptake the EPC Exos while Exos can be also uptaken in a dose- and time-dependent manner by astrocytes at the abluminal surface after exposure to ischemic conditions, leading to the suppression of lipid peroxidation and oxidative stress [165]. Similar conditions were shown in ischemic mice after injection of MSC Exos [166]. It confirmed that intracellular levels of ROS were reduced in brain ECs and these effects were more evident when miR-132-3p-loaded Exos from the same cellular source was used [166]. It should not be forgotten that juxtaposed ECs can be valid cell sources to donate Exos to adjacent ECs to modulate cell behavior and activity [167]. The works have revealed that a large number of filamentous structures namely tunneling nanotubes constitute a scaffolding network at the apicollateral sides of brain ECs to transfer Exos (Fig. 7) [167]. These data showed that both endosomal systems along with tunneling nanotube networks are involved in the Exo donation between the brain ECs. Taken together, these data indicate the potential of Exos in the modulation of EC function through the BBB under physiological and pathological conditions. Yet, the mechanism of action and various effects of Exos from different sources should be addressed.

#### Exos and myelination

Myelin is a highly technical sheath created by oligodendrocytes with distinct architecture and structure. Proteolipid proteins (PLP) and myelin basic protein (MBP) are prevalent myelin proteins [168]. Oligodendrocytes can respond to suitable physiological stimuli with an inherent ability to generate specific myelin sheath membranes and create a 3D myelin structure [169]. Even though a large number of myelin proteins were discovered more than 50 years ago, nothing is known about their 3D structure or the molecular processes behind their role in myelination. In a multilayer proteolipid membrane, molecular structure and dynamics are crucial to operating physiologically. Proteins on adjacent membranes interact to form multilayers, and proteins and lipids combine to form nanoclusters, which are the building blocks for membrane organization [170]. The membrane lipid content affects the behavior of adhesion proteins. The terrestrial nervous system's myelin, a multilayered proteolipid membrane that covers certain axons, allows nerve signals to travel salutatory. Myelin is a repeating structure composed of lipid bilayers that are closely bound and are maintained together via certain proteins. Myelin has a special lipid makeup that is high in cholesterol and poor in water [168]. Animal studies suggest that myelin proteins have similar functions even if they are genetically unrelated. Proteins with comparable activities have been localized in myelin throughout evolution to ensure stability in a range of conditions. Although having a comparable structure and function, the myelin of the CNS and peripheral nervous systems (PNS) carries distinct proteins [169]. Degenerative brain illnesses are associated with defects in myelin durability and integrity. For instance, Charcot-Marie-Tooth disease (CMT) is the most prevalent hereditary neurotoxicity, whereas multiple sclerosis (MS) is an immunological illness of the CNS in which the immune response assaults the myelin layer [171]. It has been shown that close cell–cell contact and certain molecular interactions control dynamic contact between oligodendrocytes and axons in the internodal, paranodal, and juxtaparanodal areas [172]. Schwann cells (SCs) play a significant role in regional nervous circuit axonal regeneration. SCs dedifferentiate to a progenitor-like state following nerve damage and successfully direct axons to their originally focal areas. Throughout axonal regeneration, interface and soluble mediators are involved in the crosstalk between SCs and axons. Unexpectedly, Exos from SCs significantly boost axonal regeneration in vitro and improve regeneration following sciatic nerve damage in vivo [173]. There is proof that following an axotomy, SC dedifferentiates and releases Exos that particularly internalize by DRG axons and support axonal regeneration. Typical Exos markers like CD63, Tsg101, Hsp70, Hsp90, and flotillin-1 were present in any of these Exos. Additionally, they had the p75-neurotrophin receptor (p75NTR), which is a dedifferentiated SC phenotypic feature and is often generated in response to nerve injury. Data have proved the critical role of Exos in paracrine interaction between the neuron–neuron and neuron-glia interactions within the CNS, having significant implications for myelin development and restoration [174]. MBP, PLP, 2′3'-cyclic-nucleotide phosphodiesterase (CNPase), and myelin oligodendrocyte glycoprotein are dateable in large levels in oligodendroglia Exos. Besides, myelin-specific lipids, peroxiredoxin, and heat shock proteins can be transferred via Exos [175]. Data suggest oligodendroglia Exos had favorable impacts on the neuronal pulse, mortality, and antioxidant capacity under respiratory failure. So, Exos play an important role in myelin sheath driven by neuron-oligodendrocyte contact [176]. Although the same mechanisms participate in the control of Exo secretion and myelination in both oligodendrocytes in CNS and Schwann cells in PNS, the number of Exos can differ in both cells. Like other Exo types, markers such as Hsp70, 90, Tsg101, flotillin-1, and CD63, can be detected in SC Exos. The adjacent axons' internalization of SC-derived Exos encoding CD63-GFP was initially demonstrated. Following sciatic nerve compression, these SC-derived Exos boosted the rate of axonal regeneration in dorsal root ganglion (DRG) neurons both in vitro and in vivo, demonstrating the function of Exos in axonal restoration [169]. Restoration SC-derived Exos facilitated the result of increasing neurite growth from DRG plantlets by transferring exosomal miRNA-21, concurrently with PTEN beneath and PI3K stimulation in the neurons [31]. By the downregulation of c-Jun and Sox2 transcriptional activity using retroviral transduction, it was further proven that the impact of healing SC-derived Exos is reliant on their production [124]. In line with these data, Exos play a role in axon-glia interaction and the workable differences among both Exos derived from distinctive and repair SCs suggest that the exosomal cargo content varies. This distinction clarifies the distinctive ability of restorative SCs to aid in axonal rebuilding and improved function following nerve damage [173]. The use of the caspase-3 inhibitor z-DEVD-FMK revealed that conditioned response apoptosis might be the principal process for this Exo-mediated increased cell survival. The promotion of SC injury coincides with the alteration of the exosomal profile and the release of specific markers such as the p75-neurotrophin receptor (p75NTR) [173]. This factor is associated with the dedifferentiation of SCs during post-nerve injury [177]. Molecular and cellular investigations have revealed that SCs use a calmodulin-dependent pathway in response to DRG-derived glutamate stimulus for Exo biogenesis and abscission [173]. Previously, it was demonstrated that SCs produced Exos in a calcium-dependent way in response to a glutamate stimulation generated from the DRG, increasing the function of nerve cells. Protein disulfide-isomerase A3, fibronectin, flotillin-2, the major vault protein (MVP), monocarboxylate transporter 1, neuropilin-2 (NRP2), septin-7, and syntenin-1 were all found in Exos from the treated cells of SCs [178]. These compounds are strongly connected to axon renewal. B-crystallin and galectin-1, which have anti-inflammatory properties, were also present. Bioinformatics analyses have suggested the existence of molecules involved in axon renewal, including fatty acid-binding protein, fibronectin, MVP, carboxypeptidase E, NRP2, galectin-1, and protein disulfide-isomerase B-crystallin, both of which have anti-inflammatory properties [179]. Likewise, CNS epithelial cells can alter the composition of their Exo after myelin injury [178]. The function of the GTPase RhoA, which is responsible for development cone collapse and axon retraction, is reduced by Exos, and the development cone shape is changed to a pro-regenerating pattern [171].

## Conclusion

Exos are valid cell byproduct nanoparticles with pleiotropic effects on several cell lineages. Emerging data have indicated Exos are valid de novo reservoirs with certain biomarkers for monitoring the type, intensity, and progression of pathological conditions, especially in CNS with specific molecular identities. The existence of discontinuities in the BBB barrier interface is one of the most important issues found in several CNS injuries and inflammatory responses. Exos with unique cargos can be used for the alleviation of inflammatory response and restoration of BBB cell normal activities. Of course, the type, sources, and dose of Exos for in vivo application remain to be elucidated using several future studies.

Despite the several advantages associated with the application of Exos, it seems that delivery of circulating Exos to the target sites is one of the challenging issues [180, 181]. That said, Exos lack an appropriate targeting capacity and systemic administration leads to off-target effects and accumulation in non-specific organs such as pulmonary, hepatic, and splenic tissues. Of note, allogeneic Exos are sequestrated by alloreactive T lymphocytes in lymphoid tissues [182]. These features indicate the superiority of autologous Exos over allogeneic and xenogeneic Exos for therapeutic purposes. Commensurate with these comments, the application of certain loading techniques and surface modification approaches is mandatory to increase the on-target therapeutic effects of Exos [181]. Besides these challenges, isolation, and purification of Exos using conventional and available approaches are problematic. For instance, high-rate pelleting can affect the morphology and increase the possibility of damage to the Exo membrane and protein contamination [47, 183]. It is suggested inappropriate Exo concentrations and the existence of specific factors such as tissue factor and other procoagulant factors can contribute to thrombosis and hemostatic perturbations [184]. Exos isolated from biofluids exhibits more heterogeneity with higher pro-thrombotic capacity compared to Exos purified from in vitro conditions [184]. Taken together, attempts should be concentrated to develop GMP-grade isolation and preparation technologies in terms of Exo to circumvent current limitations associated with BBB and CNS therapy.

## Data Availability

Not applicable.

## Abbreviation

AD: Alzheimer’s disease
ALS: Amyotrophic lateral sclerosis
BBB: Blood-brain barrier
BBB: Blood-brain barrier
CNS: Central nervous system
CMT: Charcot-Marie-Tooth disease
DRG: Dorsal root ganglion
ECs: Endothelial cells
EPC: Endothelial progenitor cell
Exos: Exosomes
ECM: Extracellular matrix
EVs: Extracellular vesicles
ILVs: Intraluminal vesicles
JAMs: Junctional adhesion molecules
MVP: Major vault protein
MMPs: Matrix metalloproteinases
MS: Multiple sclerosis
MVBs: Multivesicular bodies
MBP: Myelin basic protein
NRP2: Neuropilin-2
PNS: Peripheral nervous systems
PLP: Proteolipid proteins
ROS: Reactive oxygen species
SCs: Schwann cells
SEC: Size exclusion chromatography
TJs: Tight junctions
TSG101: Tumor susceptibility gene 101
VEGF: Vascular endothelial growth factor
Aβ: β-Amyloid
